# Factors associated with safe child feces disposal practices in Ethiopia: evidence from demographic and health survey

**DOI:** 10.1186/s13690-015-0090-z

**Published:** 2015-10-26

**Authors:** Muluken Azage, Demewoz Haile

**Affiliations:** Department of Public Health, College of Medicine and Health Sciences, Bahir Dar University, P.O.Box 79, Bahir Dar, Ethiopia; Department of Reproductive Health, College of Medicine and Health Sciences, Bahir Dar University, Bahir Dar, Ethiopia

**Keywords:** Child feces disposal, Ethiopia, Demographic and Health Survey, Sanitation, Diarrhea, Water Sanitation and Hygiene (WASH)

## Abstract

**Background:**

According to the WHO/UNICEF Joint Monitoring Programme (JMP) for water supply and Sanitation definition, safe child feces disposal practices include: children defecation into a latrine, disposal of child stools in a latrine or burial. Inappropriate disposal of human feces including unsafe child feces disposal facilitates the transmission of pathogens. However, the factors associated with safe child feces disposal practices have not been yet well explored in Ethiopia. This study aimed to identify factors associated with safe child feces disposal practices in Ethiopia.

**Methods:**

This study analyzed data from Ethiopian Demographic and Health Survey (EDHS) 2011. The practice of child’s feces disposal was categorized into ‘safe’ and ‘unsafe’ based on the WHO/ UNICEF JMP for water supply & Sanitation definition. Binary and multivariable logistic regression models were employed to identify factors associated with safe child feces disposal practices.

**Result:**

The prevalence of safe child feces disposal was 33.68 % (95 % CI: 32.82-34.55). In the final multivariable logistic regression model, the practice of safe disposal of child feces was significantly associated with urban residency (AOR = 1.25, 95 % CI: 1.01-1.55) and having access to an improved latrine (AOR = 1.92, 95 % CI: 1.56-2.36). Households found in the poorer, middle, richer and richest wealth quintile had (AOR = 2.22, 95 % CI: 1.70-2.89), (AOR = 2.94, 95 % CI: 2.27-3.81), (AOR = 4.20, 95 % CI: 3.42-5.72) and (AOR = 8.06, 95 % CI: 5.91-10.99) times higher odds to practice safe child feces disposal respectively as compared households from poorest wealth quintile. Mothers/caregivers with primary, secondary and higher educational status had (AOR = 1.29, 95 % CI: 1.10-1.50), (AOR = 1. 64, 95 % CI: 1.12-2.41) and (AOR = 2.16, 95 % CI: 1.25-3.72) times higher odds to practice safe child feces disposal respectively than those mothers who had no education. Those mothers/caregivers whose child was 48–59 months old had (AOR = 2.21, 95 % CI: 1.82-2.68) times higher odds to practice safe child feces disposal as compared to mothers/caregivers who had a child with age less than 12 months old. The odds of safe child feces disposal among households who had one two and three under five years old children were (AOR = 3.11, 95 % CI: 1.87-5.19),(AOR = 2.55, 95 % CI: 1.53-4.24) and (AOR = 1.92, 95 % CI: 1.13-3.24) times higher respectively than households with four and more children of under five years old.

**Conclusion:**

Only one third of the mothers practiced safe child feces disposal in Ethiopia. Being an urban resident, having a higher wealth quintile, high levels of maternal education, older child age, having a lower number of under five years old children, and the presence of an improved latrine were factors associated with safe child feces disposal practices. Therefore interventions designed to improve safe child feces disposal practices should consider those factors identified. Further research is also needed to design intervention that will aim to improve safe child feces disposal.

## Background

Inappropriate disposal of human feces, such as the practice of open defecation, facilitates the transmission of pathogens that cause enteric diseases including diarrheal diseases [1–4]. Diarrhea is one of the major public health problems worldwide, particularly for children of under five years old [1, 5, 6]. It is estimated that 1.7 billion cases of diarrhea occur every year, causing approximately 800,000 deaths among under-five children worldwide [7, 8]. Globally about 1 billion people which accounts 14 % of the global population, still engage in open defecation. About 9 % of the urban population and 34 % of the rural population in Sub-Saharan Africa practiced open defecation in 2012 [9]. Unhygienic disposal of child feces has been also reported as one of the widely practiced sanitation problem in Sub-Saharan Africa countries [10–12].

According to the WHO/ UNICEF Joint Monitoring Programme (JMP) for water supply and Sanitation definition, safe child feces disposal practices include child defecation into a latrine, disposal of child stools in a latrine or burial [13]. The improper disposal of child feces was reported as one of the factors associated with high incidence of enteric diseases [14–17]. A meta-analysis study found that unsafe child feces disposal practices such as open defecation, stool disposal in the open, stools not removed from soil, and stools seen in a household soil increased the risk of diarrheal diseases by 23 % [16]. Another study from Bangladesh found that disposal of child feces into improved latrines decreased the risk of helminthiasis by 35 % in children under two years of age [14].

Ethiopia has made a great progress in the provision of improved latrine through the implementation of health extension packages since 2003 and decreased the practice of open defecation from 61 to 39 % between 2005 and 2010 [18]. The Ministry of Health of Ethiopia, in collaboration with other stakeholders, has adopted Community-Led Total Sanitation (CLTS) to be implemented in the country through its Health Extension program [19]. CLTS is an approach which helps communities to understand and realize the negative effect of poor sanitation and empowers them to collectively find solutions to their inadequate situation. It focused on igniting a change in sanitation behavior rather than construction of toilets. CLTS targets a multitude of hygiene behaviors including safe disposal child feces. Besides safe child feces disposal, CLTS also targets ending open defecation, hygienic toilet use, hygienic food and water handling and hand washing at appropriate times [20]. CLTS aims to ignite community-wide behavior change and collective action to move the entire community toward improving sanitation together. Follow-up and monitoring activities are the most important activities for achieving an open defecation free community after implementing CLTS [20]. Ethiopian national sanitation and hygiene strategy centers on eliminating the practice of open defecation using CLTS approach [19, 21]. However, the practice of safe child feces disposal is still low in Ethiopia. Moreover factors associated with safe child feces disposal practice have not yet been well explored. Therefore, the aim of this study was to identify factors associated with safe child feces disposal practice that helps public health professionals to design effective interventions against the problem.

## Methods

### Study design and setting

This study was an in-depth secondary data analysis of a population-based cross-sectional survey of EDHS in 2011. EDHS was designed to provide population and health indicators at the national (urban and rural) and regional levels. The EDHS samples were drawn through two stages stratified clustered sampling from a total of 624 clusters (187 in urban areas and 437 in rural areas) in nine regional states in the country. Design effect was used to reduce the sampling error due to the use of a more complex and less statistically efficient design, such as multistage and cluster selection. Data from a total of 11, 654 respondents were collected and all respondents who responded for the outcome variable were included in the analysis for this study. The detailed methodology is found elsewhere [22].

### Explanatory variables

Independent variables from EDHS data set such as mother/caregiver educational level, partner educational level, age of the mother, place of residence (urban or rural), age of child, number of under five years old children, marital status, religion, and wealth index were included. The wealth index was measured using principal component analysis. Variables included in the construction of the wealth index were ownership of selected household assets, size of agricultural land, quantity of livestock and materials used for house construction. Other factors such as exposure to mass media (radio, television and newspapers), environmental health (latrine availability, drinking water supply), child diarrhea morbidity in the past two week preceding the survey, and health service related factors (visited by health workers in the past one year, visit health institution in the past one year) were included.

### Outcome measures

The outcome variable for this study was child feces disposal practices. Child feces disposal practices was assessed using WHO/UNICEF Joint Monitoring Program(JMP) for water supply and Sanitation definition by asking “The last time child passed stools (indexed for youngest under five years old child), what was done to dispose of the stools?” The list of disposal options include: did the child use the toilet or latrine, were the feces put/rinsed into the toilet or latrine, put/rinsed into a drain or ditch, thrown into garbage, buried and left in the open. Finally, child feces disposal practices were recoded into a binary outcome, “safe” (defecation into a latrine, disposal of stools in a latrine or buried) and “unsafe”(put/rinsed into a drain or ditch, thrown into garbage, and left in the open) based on WHO/UNICEF Joint Monitoring Program(JMP) for water supply & Sanitation definition [13].

### Statistical analysis

Data were analyzed by using STATA version 12 (Stata Corp, College Station, Texas, United States). We used “svy” in STATA version 12 to weight the survey data to adjust for the cluster sampling design. These sample weights were also used in order to compensate for the unequal probability of selection between the strata that has been geographically defined as well as for non-responses. A detailed explanation of the weighting procedure with all specification can be found in 2011 EDHS report page 278–279 [22]. Weighted prevalence of safe child feces disposal practice with 95 % confidence interval was done based on background characteristics of respondents.

Binary and multivariable logistic regressions models were employed to determine the factors associated with safe child feces disposal practices. A multi-collinearity test was done and variables with variance inflation factors (VIF) of greater than 10 were excluded from the multivariable analysis [23]. Those respondents with missing data were not included in the regression analysis. All variables with p-value <0.05 in binary logistic regression analysis were entered into the multivariable logistic regression model. Those variables with a p value < 0.05 in the final multivariable logistic regression model were considered as associated factors for safe child feces disposal. Both crude (COR) and adjusted odds ratios (AOR) were calculated with a 95 % confidence interval.

### Ethical statement

The data were downloaded and used after the purpose of the analysis was communicated and approved by Measure DHS. The original DHS data were collected in conformity with international and national ethical guidelines. Ethical clearance was provided by the Ethiopian Public Health Institute (EPHI) former Ethiopian Health and Nutrition Research Institute (EHNRI) Review Board, the National Research Ethics Review Committee (NRERC) at the Ministry of Science and Technology, the Institutional Review Board of ICF International, and the CDC. Written consent was obtained from mothers/caregivers and data were recorded anonymously.

## Results

Of the total 11, 654 households, 11,126 households responded to the outcome variable question in this study, which made the response rate 95.5 %. Among mothers 4.72 % reported their child used latrine for defecation while 27.84 % of children’s stools were put/rinsed into toilet/latrine. However, most children’s stools (42.01 %) were left in the open/not disposed of and 14.08 % of the children’s stools thrown into garbage. Small proportion (1.11 %) of children’s stools were buried (Table 1).

**Table 1:** Weighted prevalence of child feces disposal practice in Ethiopia, DHS 2011

Child feces disposal practices	Weighted frequency	Weighted percent
Used toilet/latrine	538	4.72
Put/rinsed in toilet/latrine	3,178	27.84
Put/rinsed into drain or ditch	394	3.45
Throw into garbage	1,607	14.08
Buried	127	1.11
Left in the open/not disposed of	4,795	42.01
Other	774	6.78

The prevalence of safe child feces disposal was found to be 33.68 % (95 % CI: 32.82-34.55). The highest prevalence of safe child feces disposal was found in Addis Ababa 74.01 % (95 % CI: 67.97-79.89) followed by South Nation, Nationalities and People regional state 47.77 % (95 % CI: 45.76-49.76). The lowest prevalence of safe child feces disposal was found in Gambella region 22.73 % (95 % CI: 11.22-38.83) followed by Amhara region 23.49 %(95 % CI: 21.88-25.17). The prevalence of safe child feces disposal was 29.81 % (95 % CI: 28.92-30.72) among rural residents. The prevalence of safe child feces disposal was 17.56 % (95 % CI: 16.14-19.05) among poorest wealth quintile households while it was 61.03 % (95 % CI: 58.69-63.35) among the richest wealth quintile households. Among those mothers who did not attended any formal education, the prevalence of safe child feces disposal was 28.34 % (95 % CI: 27.36-29.34) while it was 78.14 % (95 % CI: 70.86-83.94) among mothers who had attended higher education (Table 2).

**Table 2:** Prevalence of safe child feces disposal by background characteristics in Ethiopia: From the EDHS 2011

Background characteristics	Weighted number		Weighted proportion of safe child feces disposal (95 % CI)
	Unsafe	Safe^a^	
Region			
Tigray	493	243	32.99 (29.73-36.53)
Affar	78	38	32.55 (24.91-42.02)
Amhara	1947	598	23.49 (21.88-25.17)
Oromiya	3386	1462	30.15 (28.88-31.46)
Somali	222	121	35.26 (30.35-40.45)
Benishangul-Gumuz	78	57	42.07 (34.1-50.68)
SNNP	1245	1139	47.77 (45.76-49.76)
Gambela	27	8	22.73 (11.22-38.83)
Harari	17	10	36.84 (20.58-56.15)
Addis Ababa	54	153	74.01 (67.97-79.89)
Dire Dawa	21	16	41.86 (28.09-59.42)
Residence			
Urban	551	863	61.03 (58.47-63.55)
Rural	7,019	2,981	29.81 (28.92-30.72)
Wealth index			
Poorest	2,155	459	17.56 (16.14-19.05)
Poorer	1932	617	24.21 (22.57-25.9)
Medium	1602	762	32.25 (30.37-34.14)
Richer	1,225	979	44.41 (42.35-46.5)
Richest	655	1,026	61.03 (58.69-63.35)
Mother educational status			
No education	5,697	2,253	28.34 (27.36-29.34)
Primary	1,754	1,314	42.83 (41.09-44.59)
Secondary	84	156	64.90 (58.8-70.84)
Higher education	34	120	78.14 (70.86-83.94)
Father education			
No education	4,068	1,578	27.94 (26.77-29.11)
Primary	3,008	1,691	35.99 (34.62-37.37)
Secondary	248	308	55.31 (51.24-59.49)
Higher education	128	231	64.38 (59.28-69.18)
Age of the child			
<12 months	1766	584	24.88 (23.13-26.62)
12-23 months	1227	670	35.32 (33.19-37.49
24-35 months	1311	720	35.46 (33.41-37.57)
36-47 months	1550	788	33.70 (31.81-35.64)
48-59 months	1329	897	40.32 (38.27-42.35)
Mother’s age			
15-24	1881	836	30.77 (29.05-32.52)
25-34	3877	2063	34.73 (33.53-35.95)
≥35	1811	944	34.26 (32.51-36.05)
Number of under five old children			
One	2295	1590	40.92 (39.39-42.48)
Two	3655	1757	32.47 (31.23- 33.72)
Three	1319	423	24.28 (22.31- 26.34)
Four and above	156	30	16.32 (11.36- 21.95)
Religion			
Orthodox	2999	1324	30.62 (29.27-32.01)
Catholic	42	59	58.17 (48.63-67.73)
Protestant	1,536	1115	42.05 (40.17-43.93)
Muslim	2,806	1,263	31.05 (29.63- 32.47)
Traditional	89	29	24.66 (17.45-32.94)
Marital status			
Single	50	17	25.46 (16.05-36.78)
Married	7,034	3,592	33.81 (32.91-34.71)
Widowed	129	73	36.12 (29.73-42.94)
Divorced	356.	161	31.12 (27.26-35.23)
Water source			
Piped water	2,348	1,734	42.48 (40.96-43.99)
Other improved	672	374	35.74 (32.86-38.66)
Un improved	446	149	24.97 (21.68-28.64)
Latrine type			
Improved	402.	423	51.29 (47.86-54.68)
Unimproved	6,934	3,346	32.55 (31.65-33.46)
Reading the newspaper			
Yes	376	479	56.00 (52.61-59.26)
No	7,188	3,363	31.87 (30.99- 32.76)
Listening radio			
Yes	3,540	2,154	37.83 (36.58-39.1)
No	4,024	1,687	29.54 (28.37-30.73)
Watching TV			
Yes	2,124	1,591	42.84 (41.23-44.41)
No	5,430	2,251	29.30 (28.3-30.33)
Visited by family planning health worker in the past one year			
Yes	1,269	910.	41.78 (39.7- 43.84)
No	6,292	2,933	31.79 (30.84-32.74)
Visited health institution in the last 12 months			
Yes	2,738	1,775	39.34 (37.91-40.76)
No	4,823	2,067	30.00 (28.93-31.09)
Diarrhea in the last two weeks			
Yes	979	533	35.22 (32.87-37.69)
No	6,195	3,127	33.55 (32.59-34.5)
Total	7,570	3,844	33.68 (32.82-34.55)

^a^the sum of used toilet/latrine, put/rinsed in toilet/latrine and buried

### Factors associated with safe child feces disposal practices

In binary logistic regression analysis, from socio-demographic and economic variables: place of residence, wealth index, mother and partner education status, marital status, number of under five years old children, child age and religion were factors associated with safe child feces disposal practices. Listening to radio, watching television and reading newspaper at least once a week, being visited by family planning health workers in the past one year, and visited health institutions in the past one year were also significantly associated with safe child feces disposal practices. Water supply (piped water supply) and an improved latrine had a statistically significant association with safe child feces disposal practices in binary logistic regression model (Table 3).

**Table 3:** Regression analysis of factors associated with safe child feces disposal in Ethiopia, DHS 2011

Variables	^a^COR (95 % CI)	^a^AOR (95 % CI)
Place of residence		
Rural	1.00	1.00
Urban	3.68 (3.28-4.13)	1.25 (1.01-1.55)
Household wealth index		
Poorest	1.00	1.00
Poorer	1.50 (1.31-1.71)	2.22 (1.70- 2.89)
Middle	2.23 (1.96-2.55)	2.94 (2.27-3.81)
Richer	3.75 (3.29-4.28)	4.20 (3.42- 5.72)
Richest	7.35 (6.39-8.46)	8.06 (5.91-10.99)
Child age		
<12 months	1.00	1.00
12-23 months	1.65 (1.44-1.88)	1.66 (1.37-2.04)
24-35 months	1.66 (1.46-1.89)	1.64 (1.35-2.00)
36-47 months	1.54 (1.35-1.74)	1.60 (1.32-1.94)
48-59 months	2.04 (1.80-2.31)	2.21 (1.82-2.68)
Mother’s age		
15-24	1.00	1.00
25-34	1.20 (1.09-1.32)	1.10 (0.94-1.29)
> = 35	1.17 (1.05-1.31)	1.18 (0.99- 1.42)
Number of under five old children		
One	3.55 (2.39-5.26)	3.11 (1.87-5.19)
Two	2.46 (1.67-3.64)	2.55 (1.53-4.24)
Three	1.64 (1.09-2.46)	1.92 (1.13- 3.24)
Fourth and above	1.00	1.00
Religion		
Orthodox	1.00	1.00
Catholic	3.15 (2.11- 4.70)	1.53 (0.83, 2.81)
Protestant	1.64 (1.49- 1.82)	1.18 (0.93 - 1.50)
Muslim	1.02 (0.93-1.11)	1.15 (0.96- 1.38)
Traditional	0.74 (0.49- 1.13)	0.98 (0.39 - 2.45)
Mother’s education		
No education	1.00	1.00
Primary	1.89 (1.74-2.07)	1.29 (1.10-1.50)
Secondary	4.68 (3.57- 6.12)	1.64 (1.12-2.41)
Higher	9.04 (6.15-13.29)	2.16 (1.25-3.72)
Partner’s education		
None	1.00	1.00
Primary	1.45 (1.33- 1.58)	1.75 (0.65-1.86)
Secondary	3.19 (2.67- 3.81)	1.12 (0.85-1.48)
Higher	4.66 (3.73- 5.83)	1.97 (0.67-2.41)
Current marital status		
Single	1.00	1.00
Married	1.11 (0.96-1.29)	1.23 (0.97-1.56)
Widowed	0.70 (0.55-0.87)	1.00 (0.71-1.42)
Divorced	1.57 (1.12-2.20)	1.15 (0.67-2.00)
Source of water supply		
Piped water	2.22 (1.82- 2.70)	0.99 (0.78-1.26)
Other improved water	1.67 (1.34- 2.09)	1.11 (0.86-1.45)
Unimproved	1.00	1.00
Latrine		
Improved	2.18 (1.89-2.51)	1.92 (1.56-2.36)
Unimproved	1.00	1.00
Listening radio		
Yes	1.45 (1.34-1.57)	
No	1.00	
Watching Television		
Yes	1.81 (1.67-1.96)	
No	1.00	
Reading news letter		
Yes	2.72 (2.36- 3.13)	
No	1.00	
Visited by Health extension worker in the past one year		
Yes	1.53 (1.39- 1.69)	
No	1.00	
Visit health institutions in the past one year		
Yes	1.51 (1.39- 1.63)	
No	1.00	
Children with diarrhea in the past two weeks		
Yes	1.00	
No	1.09 (0.97- 1.21)	

^a^COR = crude odds ratio, AOR = Adjusted odds ratio

Variables: Listening to radio, reading newspaper, watching television, being visited by family planning worker in the last 12 months, visited health institutions in the last one year were excluded from the final multivariable logistic regression model because of collinearity. In multivariable logistic regression analysis place of residence, wealth index, mother’s education, number of under five years old children, child age and improved latrine were significantly associated with safe child feces disposal practices.

The odds of practicing safe child feces disposal were (AOR = 1.25, 95 % CI: 1.01-1.55) times higher among urban residents as compared to rural residents. Households found in the poorer, middle, richer and richest wealth quintile had (AOR = 2.22, 95 % CI: 1.70-2.89), (AOR = 2.94, 95 % CI: 2.27-3.81), (AOR = 4.20, 95 % CI: 3.42-5.72) and (AOR = 8.06, 95 % CI: 5.91-10.99) times higher odds to practice safe child feces disposal respectively as compared to households from the poorest wealth quintile.

Those mothers/caregivers whose child age was in the range of 12–23, 24–35, 36–47, and 48–59 months had (AOR = 1.66, 95 % CI: 1.37-2.04), (AOR = 1.64, 95 % CI: 1.35-2.00), (AOR = 1.60, 95 % CI: 1.32-1.94), (AOR = 2.21, 95 % CI: 1.82-2.68) times higher odds of practicing safe child feces disposal respectively as compared to those mothers/caregivers who had child with age less than 12 months.

The odds of practicing safe child feces disposal among households who had one under five years old child, two and three under five years old children were (AOR = 3.11, 95 % CI: 1.87-5.19), (AOR = 2.55, 95 % CI: 1.53-4.24) and (AOR = 1.92, 95 % CI: 1.13-3.24) times higher than households who had four and above children respectively. Mothers/caregivers with primary, secondary and higher education level had (AOR = 1.29, 95%CI: 1.10-1.50), (AOR = 1. 64, 95 % CI: 1.12-2.41) and (AOR = 2.16, 95 % CI: 1.25-3.72) times higher odds to practice safe child feces disposal respectively than those mothers/caregivers with no education. Those mothers/caregivers who were from households with an improved latrine had (AOR = 1.92, 95 % CI: 1.56-2.36) times higher odds to practice safe child feces disposal than those who had unimproved latrine (Table 3).

## Discussion

Only one third of the mothers/caregivers practiced safe child feces disposal in Ethiopia. This mean that two-thirds of the population was at increased risk of pathogen exposure from contaminated environment with child feces besides other contaminants. Safe child feces disposal practice may be particularly important in prevention of fecal-oral transmission as children are more susceptible to these diseases and are often defecating in areas where other children could be exposed. As evidenced by researchers, usually children open defecate inside the home or in the compound near the house so that its proximity to households may increase the risk compared to the more typically distant open defecation sites [24]. The practice of unsafe child feces disposal contaminates the surrounding environment with human excreta which carries many infectious organisms that can cause enteric diseases such as childhood diarrhea [1–4].

The prevalence of safe child feces disposal found in this study is relatively similar with prevalence which is reported from Madagascar (38 %) [10]. However the prevalence of safe child feces disposal was 67 % in Zambia [25], 70 % in Kenya [11], 75 % in Uganda [26] and 79 % in Malawi which are higher as compared to this study [27]. In contrast very high prevalence of (81.4 %) unsafe child disposal was reported from small scale study conducted in Rural Orissa, India. Our study found that the most common type of unsafe child feces disposal method was left child feces in the open or not disposed. Many studies from different part of the world reported that leaving child feces in the open or not disposed is a common child feces disposal practices [24, 28, 29].

The highest prevalence of safe child feces disposal was found in Addis Ababa. This could be due to the fact Addis Ababa is the capital city where the residents are relatively educated as compared to other part of the country and have relatively better sanitation facilities. Southern Nation, Nationalities and Regional state had relatively high prevalence of safe child feces disposal as compared to other regions. This region is the first region which has pioneered Community Led Total Sanitation in Ethiopia. Reports from Southern regions showed that access to sanitation reaches to 75 %, the highest of any region in Ethiopia [30, 31].

Place of residence was associated with safe disposal of child feces. The odds of practicing safe child feces disposal were higher among urban residents. This finding is consistent with a study from Kenya [32]. The possible justification could be urban residents might have better interventions on water, sanitation and hygiene than rural areas which in turn can influence hygienic behavior. WHO/UNICEF reported that there is a big gap between urban and rural residents in access to improved sanitation and open defecation practice. Rural areas, especially those remote and difficult-to-reach areas have markedly lower access to improved water and sanitation [9]. In Ethiopia, there is urban rural disparity regarding sanitation coverage [33], toilet facility and access to safe drinking water [34]. The desire to conform to specific social norms/expectations relating to hygiene behavior may also, in part, explain the urban–rural disparities in practicing safe child feces disposal as evidenced by the study from Burkina Faso [35].

This study revealed that the odds of practicing safe disposal of child feces were increased with increased level of mothers’ education. A Kenyan study showed that increased levels of education of the mother was associated with increased safety in disposal of children’s stools [32]. A similar finding was reported in other developing countries which revealed that those mothers who had completed at least primary education have better hygienic behavior and child care practices [36–38]. Educated mothers are more likely to understand causes of childhood illness [39] so that practiced more hygienic behavior to protect their child from illness.

Household socioeconomic status was reported as one of the factors that determine the practice of essential hygienic behavior [37, 40]. This study also found that households from a higher wealth quintile were more likely to practice safe disposal of child feces than those households from the poorest wealth quintile. Those households with better wealth status had more likely improved sanitation for a better standard of living, that might motivate them to dispose of child feces safely [41].

In this study, age of children was also one of the factors associated with safe child feces disposal. As age of the child increases the likelihood of practicing safe disposal of child feces becomes higher. This finding is consistent with other studies which revealed that children with age three and above three years old were less likely to practice open defecation [29, 42]. Stool of young children are considered as harmless, or at least less harmful than those of adults, because they are smaller, their feces smell less, and contain less visual food residues [16, 43]. However, when the age of the child becomes older, the child feces would be characterized by bad smell and visual food residues which make the feces more disgusting [16]. This could be the possible justification why stools of older children disposed safely as compared to stools of younger children. The other justification could be those older children could able to defecate independently into latrine [16] . This study also found that having lower number of under five years old children in a household associated with higher odds of practicing safe child feces disposal. This finding coincides with another study [37]. Having lower number of under five years old children might reduce the burden on mothers/caregivers and they would have time to practice better hygiene behavior. Mothers who had high work load had poor hygienic behavior for under five years old children [44].

Those mothers/caregivers from households with an improved latrine had higher odds to practice safe disposal of child feces. A similar finding was reported from South Africa [41]. Owning latrine is a necessary requirement to adopt safer child feces disposal practices [24, 45]. Another study revealed that ownership of physical infrastructure of improved sanitation can motivate people to adopt safe hygienic practices. However, improvement and presence of physical infrastructure alone is not sufficient to ensure adoption of safe hygienic practices [41].

Availability of improved water supply was not significantly associated with the practice of safe child feces disposal. However, this finding contradict with another study which showed that presence of piped water in the compound was found as one of the independent predictor of practicing safe disposal of child feces [35]. This finding of our study might implicates that having improved water source alone is not sufficient to ensure safe child feces disposal practices in Ethiopia.

This study has limitations. This study shares the limitation of cross sectional study design, that is unable to establish cause and effect relationship [46]. Since we used secondary data, all variables that influence the practice of safe disposal of child feces are not exhaustively included in the analysis [46]. For instance, the perception and knowledge of mothers’ about consequence of child feces was not included in the survey. The other limitation of this study was the social desirability bias that decreases the likelihood that people will report poor child feces disposal practices [47, 48]. Finally some of the regions had small a sample size, which questions the accuracy of prevalence estimates [49] per region, so that it should be interpreted with caution.

## Conclusions

The practice of safe child feces disposal was low in Ethiopia. There is regional variation in prevalence of safe child feces disposal. Being an urban resident, higher wealth index, higher maternal education, older child age, having lower number of under five years old children and presence of an improved latrine were factors associated with safe child feces disposal practices. Interventions designed to improve the practices of safe child feces disposal should consider those factors identified. Further research is also needed to design intervention that will aim to improve safe child feces disposal.

## Abbreviations

AOR: Adjusted Odds Ratio
CLTS: Community-Led total sanitation
COR: Crude Odds Ratio
DHS: Demographic and Health Survey
EDHS: Ethiopian Demographic and Health survey
UNICEF: United Nations Emergency Children’s Fund
WASH: Water, sanitation and Hygiene, WHO, World Health Organization
